# Dataset of a national survey on online gambling activities among young people in Portugal.

**DOI:** 10.12688/f1000research.152851.2

**Published:** 2025-03-07

**Authors:** Ana Rita Farias, Ana Cristina Antunes

**Affiliations:** 1 HEI‐Lab: Digital Human‐Environment Interaction Labs, Lusófona University, Lisbon, Lisbon, Portugal; 2 School of Communication and Media Studies (ESCS), LIACOM, Polytechnic Institute of Lisbon, Lisbon, Lisbon, Portugal

**Keywords:** Online Gambling Behavior; Young Gamblers, Antecedents, Consequences, and Sociodemographic Factors

## Abstract

**Background:**

Recent advancements in online gambling have significantly increased the popularity and participation in gambling activities among the general population, specifically the young generations. These changes are reshaping gambling behaviors, attracting a growing number of enthusiasts. This paper describes a dataset that maps online gambling activities among young individuals in Portugal, providing insights into their gambling prevalence, habits, behaviors, preferences, and potential antecedents and consequences of these activities.

**Methods:**

A survey was conducted with a representative sample of 1,993 young people in Portugal, aged between 18 and 34, to gather data on their activities. This methodology involved a quantitative telephone survey conducted in March and April 2023, utilizing quota sampling to ensure representation across various regions. The data collection process employed Computer Assisted Telephone Interviewing (CATI) and involved rigorous quality control measures to ensure accuracy and reliability.

**Conclusion:**

The dataset generated from this survey provides valuable insights into the patterns of online gambling activities among young Portuguese individuals. It allows researchers to explore potential risk factors, including gambling-related harm, and to understand the sociodemographic factors influencing gambling behaviors. The findings can inform interventions and policies aimed at mitigating the negative consequences of online gambling among youth.

## Introduction

The digital world has optimized numerous daily tasks, such as grocery shopping and communication. However, it has also introduced new problems or new forms of old problems. One industry significantly affected by digital transformation is gambling, and the online gambling market is growing worldwide, driven by technological advancements and increasing consumer confidence in online monetary transactions. According to Statista (2024), the revenue of the online gambling market is projected to reach US$100.90bn in 2024, with an annual growth rate (2024-2029) of 6.20%, resulting in a projected market volume of US$136.30bn by 2029, while the number of users is expected to reach 281.3 million by 2029. Notably, online gambling is currently the fastest-growing form of gambling.

The easy access and convenience of online gambling, which can be done anywhere, at any time, and for an unlimited duration, coupled with the increasing availability of digital platforms, the low costs and incentives of online gambling, and the possible anonymity, has led to a rise in the number of people engaging in these forms of gambling for money. ^1^ These trends have raised concerns among various professionals, including educators and healthcare providers, who are increasingly worried about the potential negative effects of online gambling, such as financial problems and impacts on mental health. ^2^^,^^3^ Indeed, when compared with land-based gambling, online gambling has a greater addictive potential due to a series of situational and structural aspects, such as availability, accessibility, immediacy of reinforcement, or speed and frequency of gambling. ^4^^,^^5^ Additionally, it is associated with a range of negative outcomes that make it potentially harmful to individuals’ financial, physical, and emotional well-being, as well as to their families and society as a whole. ^2^^,^^3^ Moreover, despite clear age restrictions, literature suggests that the prevalence of online gambling activities is significantly higher among younger age groups compared to adults. ^6^ Understanding this issue among young people is crucial, as these new forms of online gambling seem particularly enticing to this population.

To address the urgent need for understanding and mapping online gambling activities among Portuguese youth, this research aims to shed light on this phenomenon within this less-studied generational cohort. A telephone survey was conducted in March and April 2023, targeting a random sample of 1,993 young individuals aged 18 to 34, residing in Portugal.

## Methods

This project, which intends to map online gambling activities and examine potential antecedents and outcomes among young individuals in Portugal, received data collection support from GfK.

This quantitative study included both male and female participants, aged 18 to 34, living in Portugal. The sample consisted of 1,993 participants, divided into two sub-samples proportionately distributed across key regions in Portugal ( Table 1). Sub-sample 1 investigated the possible antecedents, while Sub-sample 2 examined the possible consequences. This division enabled a more detailed analysis of cause-effect relationships without overloading individual respondents with an excessively long questionnaire, reducing survey fatigue and increasing response quality. Respondents were selected using the quota sampling method, incorporating variables such as gender, age (categorized into 2 groups), and region (using the Portuguese NUTS II regions). Quota sampling intends “to approximate the results that would be obtained with probabilistic samples” (Romero & Bologna, 2013, p. 288), ^7^ thus reproducing the characteristics of the study population. Households were chosen by randomly generating fixed and mobile telephone numbers, ensuring representation across the different Portuguese regions based on the initial matrix. The random generation of numbers followed assigned prefixes specific to each region and operator.

**Table 1.** Sub-samples distribution among regions.

Key-regions in Portugal	Sub-sample 1 (SS1)	Sub-sample 2 (SS2)
North	357	356
Center	202	204
Lisbon	294	304
Alentejo	62	63
Algarve	41	45
Azores	25	25
Madeira	26	24
Total	1007	1021

The pilot study, conducted with six participants aged 18-34, aimed to assess survey question clarity, estimate interview duration to maintain engagement, identify technical issues with the Computer Assisted Telephone Interviewing (CATI) script, and evaluate the logical flow of questionnaire items. Based on participant feedback, minor wording adjustments were made to improve clarity, and the estimated duration was confirmed to be within an acceptable range, leading to the development of the final questionnaire.

Data was collected through telephone interviews utilizing the CATI system, supported by a questionnaire developed by the research team. The fieldwork took place between January 28th and March 25th, 2023, with a team of 47 interviewers trained by GfK Metris. Interviews were conducted on weekdays from 5 PM to 10 PM and on weekends from 11 AM to 10 PM, covering a range of suitable time slots for data collection.

### Quality control

Comprehensive quality control measures were implemented to ensure the accuracy and reliability of the data. Interviewers underwent thorough training, and we limited the inclusion of new interviewers to 25% of the total interviews. Additionally, interviews were distributed among different interviewers in each region to avoid concentration and potential bias.

The CATI system enabled the automatic validation of the data file at multiple levels, including a response code validation for each question and ensuring a logical flow between questions, such as skips and filters, to maintain the questionnaire’s structure integrity. A field technician from Metris GfK supervised the interviewers, closely monitoring adherence to household and respondent selection criteria, overseeing interview conditions and duration, and providing on-site support when needed. Errors or missing information in the survey were reviewed by the IT Department, which determined appropriate procedures to rectify issues, such as contacting respondents for missing data or voiding interviews with abnormal non-response rates. An abnormal non-response rate was determined based on a comparative assessment of the proportion of unanswered questions relative to the total number of items in the questionnaire. Cases in which the extent of missing data compromised the validity or interpretability of the responses were classified as exhibiting an excessively high non-response rate, warranting the exclusion of the interview from the final dataset. The company systematically reviewed all questionnaires to identify errors or missing information. Each case was assessed individually, with possible actions ranging from re-contacting participants to retrieve missing data to canceling interviews altogether if the non-response rate was deemed excessively high.

Additionally, a separate supervision process involved recontacting at least 10% of each interviewer’s respondents to ensure data accuracy and consistency. Open-ended questions were transcribed using CATI software (e.g. https://www.b2binternational.com/research/methods/faq/what-is-cati/), converted into numeric data, capturing 100% of the responses. This facilitated the implementation of coding plans specific to each question, ensuring proper analysis and interpretation of qualitative data.

Data coding of all variables was conducted using SPSS Statistics Version 28.0.1.0. After the coding process and comprehensive validation of the computer file, the data were ready for tabulation and further analysis using dedicated software. These measures ensured the overall quality and reliability of the collected data, enabling insightful findings on online gambling behaviors and meaningful conclusions on their predictors and consequences.

### Measures

The questionnaire is structured in four sections: after an initial screening, there is a section intended to map gambling activities in general, followed by a focus on online gambling activities, habits, preferences and problem gambling, while the third section is designed to gather information on gambling antecedents and consequences and the final section obtains demographic information of the respondents.

A)
*Initial screening* :
The initial screening measure aims to gain a preliminary understanding of participants’ gambling involvement and establish a baseline for further assessment. The key measure is *Gambling Participation* which determines whether the participant has ever engaged in any form of gambling (either online or offline). This essential question is posed with two response options: “1 - Yes” for those who gambled at least once during their lifetime and “2 - No” for those who have not. By identifying participants with prior gambling experience, researchers can segment the sample, which serves as a crucial step in understanding the dynamics of participation and involvement with gambling activities.
B)
*Main measures across the two samples*
  1) *Gambling activities map*
The following set of measures is designed to provide a comprehensive assessment of participants’ gambling experiences, habits, and spending patterns.
    • *Gambling Experience Offline/Online Measure*: Investigates whether participants have ever bet money on offline or online games using a dichotomous question (1 - Yes; 2 - No).
    • *Gambling Frequency Offline/Online Measure*: A single close-ended question tracks the frequency of participants’ gambling activities over their lifetime. It records the number of gambling occasions both online and offline, utilizing a frequency scale ranging from 1 (never) to 7 (41 or more occasions).
    • *Gambling Participation Past 12 Months and Past 3 Months Offline/Online Measures*: Adapted from Canale et al. (2016), ^8^ two close-ended questions gather information on the number of times participants engaged in online or offline gambling activities during the previous 12 months and the previous 3 months, respectively. Responses are recorded on a 5-point frequency scale, ranging from 1 (never) to 7 (41 or more occasions).
    • *Gambling Frequency per Game Types in the Last 12 Months and Last 3 Months Measure*: Based on Canale et al. (2016), ^8^ this measure assesses the frequency of participation in 11 different gambling activities, such as slot machines, Euromillions, bingo, instant lottery, sports betting, and poker. The gambling frequency on these different game types is measured using a 7-point frequency scale ranging from 1 (never) to 7 (41 or more occasions).
    • *Gambling Average Monthly Expenditure Measure*: Derived from Hubert (2015), ^9^ this close-ended question measures participants’ gambling expenditures in a typical month during the year preceding data collection. Participants are asked to indicate their monthly spending amount using a 6-point scale ranging from 1 (20€ or less) to 6 (100€ or more).
    • *Gambling Spending Typical Month Measure*: This open-ended question requires participants to specify the exact amount they spend in a typical month on both online and offline gambling activities.
  2) *Online Gambling Activities Map*
This set of measures is designed to explore various aspects of online gambling behavior and problematic gambling tendencies:
    • *Start Browsing Age*: assesses, by an open question, the age at which participants began browsing independently the internet.
    • *Device Usage Frequency Measure:* This measure assesses the frequency of device usage for online gambling, including personal computers, smartphones, and gaming consoles. Participants are asked to report how often they use for each device on a 5-point scale ranging from 1 (never) to 5 (always).
    • *Gambling location frequency:* This measure explores the frequency of online gambling in various locations, such as home, work, university/school, and outdoors. Participants are asked to indicate how often they gamble in each location using a 5-point scale ranging from 1 (never) to 5 (always).
    • *Online Betting Time Preference:* Assesses, through a single question, the preferred time of day for online betting. Participants are asked to select their preferred time from four options: morning, afternoon, night, and dawn.
    • *Online Gambling Attraction Levels:* An 11-item scale adapted from Hubert (2015) ^9^ assesses participants’ attraction to various aspects of online gambling, such as anonymity, convenience, diversity of games, and connection with others. Each item is rated on a 5-point scale, ranging from 1 (not at all attractive) to 5 (very attractive).
*
Gambling-related Problem:* Gambling problems were assessed using a version of the DSM-IV pathological gambling criteria (American Psychiatric Association, 2000), adapted from Canale et al. (2016). ^8^ Seven gambling-related problems (e.g., problems with spouse/partner and/or other people, financial problems, work-related problems) experienced in the previous 12 months are assessed. To go beyond the mere presence or absence of a symptom/problem, participants are asked to indicate the frequency that they experienced each symptom, using a 5-point frequency scale from 1 (Never) to 4 (Always).
C)
*Specific measures s1 (Antecedents) and s2 (consequents)*
  1) *Mapping antecedents (s1):*
This set of measures is designed to assess participants’ gambling antecedents comprehensively:
    • *Gambling Motives measure*: A 25-item measure adapted from Hubert (2019) ^10^ is used to assess peoples’ motives for gambling. Participants responded by indicating on a 7-point scale (1 being ‘does not apply to me at all’ and 7 ‘applies totally to me’), how much each motive could be applied to them.
    • *Gambling Advertising measure*: A 10-item scale adapted from Noble et al. (2022) ^11^ measures the perceived exposure to several types of gambling advertising and promotions. Participants are asked to indicate whether they had been aware of ads or promotions for gambling in several media (e.g., ‘Ads on TV’, ‘Ads on social media’, ‘Pop-ups on websites’, ‘Celebrities promoting gambling’) in the previous 30 days, using a 5-point frequency scale ranging from 1 (never) to 5 (always).
  2) *Mapping consequents (s2):*
This set of measures is designed to comprehensively assess the participants’ main gambling consequences:
    • *Consequences for health and well-being *: The Short Form Health Survey (SF-12) by Ware et al. (1996) ^12^ is used to assess overall health and well-being across eight domains, including physical functioning, role limitations due to physical and emotional problems, bodily pain, general health perceptions, vitality, social functioning, and mental health. It is widely used in clinical and research settings to provide a quick, reliable assessment of physical and mental health status.
    • *Consequences Due to Gambling Measure*: All 24 items of this scale adapted from Hubert (2015) ^9^ concern several consequences that can be derived from gambling (e.g., financial, for social and/or family life, on the professional sphere or for psychological and/or physical health). Participants are asked to rate the extent and severity of these various gambling-related consequences on a 6-point scale from 0 (not at all serious) to 5 (extremely serious). Items include “I couldn’t concentrate at work,” “At home, I was only physically present,” “It took up too much of my time,” and “I lost job or professional, social, or marital opportunities.”
D)
*Socio-demographics:*

The socio-demographic measures collect essential personal and household information from respondents (incl. gender; age; years of formal education completed; parents’ education levels; marital status; employment status; average weekly and monthly disposable income; and household members).

### Dataset validation

n/a

### Ethical considerations and consent to participate

The study adhered to the Declaration of Helsinki and received approval from the Ethics Committee “Comissão De Ética E Deontologia Para A Investigação Científica (CEDIC)” of Lusófona University (Protocol code: CEDIC-2023-02-08; Approval Date: February 1, 2023). Before conducting the interviews, participants were informed about the general objective of the study and verbal consent was obtained. Verbal consent was deemed appropriate due to the data collection methodology, which involved telephone interviews.

## Data Availability

The data presented are openly available at OSF repository https://osf.io: National survey on online gambling activities among young people in Portugal. ( https://doi.org/ 10.17605/OSF.IO/JEK2T ) ^13^ Data are available under the terms of the Creative Commons Attribution 4.0 International license (CC B4.0).
