# Supplementary material for: Dataset of a national survey on online gambling activities among young people in Portugal
Source: F1000Res. 2025 Mar 7;13:893. Originally published 2024 Aug 6. [Version 2] doi: 10.12688/f1000research.152851.2 (PMC12374148; doi:10.12688/f1000research.152851.2)
Supplement: Peer review history

# Reviewer report: Reviewer response for version 2

**DOI:** 10.5256/f1000research.178023.r397748

**Published:** 2025-08-22

**Authors:** Eva Grosemans

**Affiliations:** 1 Katholieke Universiteit Leuven, Leuven, Belgium

Thank you for this very useful dataset! I have a few, very small remarks.

 I think it would be useful to already add data on the gender split, as well as on the mean age and two age groups (for the whole dataset and/or for the subsets).

 What was the average duration of the interviews?

 Did participants receive a definition and/or examples of gambling?

 I think it might be useful to add some examples of gambling motivations in the methods section.

Are sufficient details of methods and materials provided to allow replication by others?

Yes

Is the rationale for creating the dataset(s) clearly described?

Yes

Are the datasets clearly presented in a useable and accessible format?

Yes

Are the protocols appropriate and is the work technically sound?

Yes

Reviewer Expertise:

Gambling, young adults

I confirm that I have read this submission and believe that I have an appropriate level of expertise to confirm that it is of an acceptable scientific standard.

# Reviewer report: Reviewer response for version 2

**DOI:** 10.5256/f1000research.178023.r394529

**Published:** 2025-08-14

**Authors:** Ståle Pallesen

**Affiliations:** 1 Department of Psychosocial Science, University of Bergen, Bergen, Hordaland, Norway; 2 Haukeland University Hospital, Bergen, Norway

This paper presents a dataset investigating online gambling behavior and various correlates among young people in Portugal as well as potential antecedents and consequences of such gambling.

  

1. Abstract: I would like to see some more results presented in the abstract (e.g., sample size broken down on the two subsets as well as overall response rate).
2. Introduction: The introduction should maybe end with some clearly and explicitly formulated research questions – as of now it is just stated that there is “an urgent need to understand…”
3. It is stated that the division into to samples (two sets of questionnaire) – was among other motivated by being able to investigate cause-effect relationship. However, as this study is based on a cross-sectional design cause-effect relationships (nor even directionality) cannot be elucidated based on such a design.
4. To me it’s very unclear whether the sample is representative of the population or not. If quota sampling was used it means that the quotas reflect the population on some variables, however selection of persons into the quotas is normally based on convenience sampling. Please elaborate.
5. If the phone numbers were randomly generated, how could only persons 18-34 years been reached? What happened if a call was directed to a household with more than one person 18-34 years of age?
6. How was missing data handled statistically (e.g., imputation)?
7. What was the response rate? This crucial information is currently lacking.
8. I do acknowledge the usefulness of screening questions – however the authors run a risk of producing underreporting, as many of those who don’t endorse any type of gambling, could have endorsed if provided with a clue/priming (e.g., a list a potential gambling forms, such as lottery, poker with friends etc.). This may be mentioned as a limitation.  
9. The response alternatives on a 7-point scale, where the last alternative is “41 or more occasions” seems to reflect data on an ordinal scale. Please state if this is correct.
10. In many instances the authors refer to scales they have adapted from other (such as Canale et al., 2016, Hubert, 2015, and Noble et al., 2022). The readers need to be informed exactly what the adaptions implies – otherwise the research is not replicable. I suggest that the authors make the whole questionnaire available in an appendix.

Are sufficient details of methods and materials provided to allow replication by others?

No

Is the rationale for creating the dataset(s) clearly described?

Partly

Are the datasets clearly presented in a useable and accessible format?

Partly

Are the protocols appropriate and is the work technically sound?

Partly

Reviewer Expertise:

Gambling/gaming and sleep

I confirm that I have read this submission and believe that I have an appropriate level of expertise to confirm that it is of an acceptable scientific standard, however I have significant reservations, as outlined above.

# Reviewer report: Reviewer response for version 2

**DOI:** 10.5256/f1000research.178023.r369955

**Published:** 2025-03-19

**Authors:** Tiange Xu

**Affiliations:** 1 University of Nevada, Las Vegas, NV, USA

No further comments to make on this revised article.

Are sufficient details of methods and materials provided to allow replication by others?

Partly

Is the rationale for creating the dataset(s) clearly described?

Yes

Are the datasets clearly presented in a useable and accessible format?

Yes

Are the protocols appropriate and is the work technically sound?

Partly

Reviewer Expertise:

Problem gambling

I confirm that I have read this submission and believe that I have an appropriate level of expertise to confirm that it is of an acceptable scientific standard.

# Reviewer report: Reviewer response for version 1

**DOI:** 10.5256/f1000research.167658.r332353

**Published:** 2024-10-31

**Authors:** Tiange Xu

**Affiliations:** 1 University of Nevada, Las Vegas, NV, USA

This manuscript describes a dataset on online gambling behaviors among Portuguese youth (18-34 years old), collected through telephone surveys with 1,993 participants.

 The study aims to understand gambling prevalence, habits, behaviors, and their antecedents/consequences. The data was collected using CATI methodology with robust quality control measures.

**Rationale for dataset creation:** The authors clearly establish the importance of studying online gambling among youth, citing relevant statistics and highlighting the growing concerns around this issue.

**Protocols and Technical Soundness:** While the overall methodology is sound, I have a few concerns: 1) Could you clarify how you handled non-response rates? What was considered an "abnormal" non-response rate? 2) The split into two sub-samples needs more explanation—what was the rationale behind this division?

**Methods and Materials for Replication:** I appreciate the detailed description of the survey methodology, but a few elements are missing: 1) The coding plans mentioned for open-ended questions should be described; 2) Could you specify the software used for data analysis? 3) Please provide more details about the pilot phase—like, how many participants were involved, and what specific changes were made based on the pilot?

Are sufficient details of methods and materials provided to allow replication by others?

Partly

Is the rationale for creating the dataset(s) clearly described?

Yes

Are the datasets clearly presented in a useable and accessible format?

Yes

Are the protocols appropriate and is the work technically sound?

Partly

Reviewer Expertise:

Problem gambling

I confirm that I have read this submission and believe that I have an appropriate level of expertise to confirm that it is of an acceptable scientific standard, however I have significant reservations, as outlined above.

# Response

**Published:** 2025-02-06

**Authors:** Ana Rita Farias

**Affiliations:** Lusofona University, Portugal

Dear Reviewer,

 We sincerely appreciate the time and effort you have taken to review our manuscript. Your insightful comments have helped us refine our work, and we have carefully addressed each of your concerns. Below, we provide detailed responses to your queries and describe the corresponding revisions made to our manuscript.

**1. Handling of Non-Response Rates**

**Reviewer’s Comment:** Could you clarify how you handled non-response rates?

**Response:** The data collection and handling of non-responses were managed by GfK Metris Portugal. The company systematically reviewed all questionnaires to identify errors or missing information. Each case was assessed individually, with possible actions ranging from re-contacting participants to retrieve missing data to canceling interviews altogether if the non-response rate was deemed excessively high. We have clarified this in the revised manuscript by integrating the following text to addressed this concern:

*“Cases in which the extent of missing data compromised the validity or interpretability of the responses were classified as exhibiting an excessively high non-response rate, warranting the exclusion of the interview from the final dataset. The company systematically reviewed all questionnaires to identify errors or missing information. Each case was assessed individually, with possible actions ranging from re-contacting participants to retrieve missing data to canceling interviews altogether if the non-response rate was deemed excessively high.”*

**2. Definition of Abnormal Non-Response Rate**

**Reviewer’s Comment:** What was considered an "abnormal" non-response rate?

**Response:** An abnormal non-response rate was determined based on a comparative assessment of the proportion of unanswered questions relative to the total number of items in the questionnaire. Cases in which the extent of missing data compromised the validity or interpretability of the responses were classified as exhibiting an excessively high non-response rate, warranting the exclusion of the interview from the final dataset. This information has been explicitly stated in the revised manuscript.

*“An abnormal non-response rate was determined based on a comparative assessment of the proportion of unanswered questions relative to the total number of items in the questionnaire.”*

**3. Rationale for Sub-Sample Division**

**Reviewer’s Comment:** The split into two sub-samples needs more explanation—what was the rationale behind this division?

**Response:** The dataset was divided into two sub-samples to allow a more focused exploration of antecedents and consequences of gambling behaviors:

**a) Sub-sample 1 (SS1):** Investigated potential antecedents of gambling behavior, such as exposure to gambling advertisements, motives for gambling, and early experiences.

**b) Sub-sample 2 (SS2):** Focused on gambling consequences, such as financial difficulties, social and psychological effects, and health impacts. This division enabled a more detailed analysis of cause-effect relationships without overloading individual respondents with an excessively long questionnaire, reducing survey fatigue and increasing response quality. This explanation has now been included in the revised manuscript as follows.

*“The sample consisted of 1,993 participants, divided into two sub-samples proportionately distributed across key regions in Portugal ( Table 1). Sub-sample 1 investigated the possible antecedents, while Sub-sample 2 examined the possible consequences. This division enabled a more detailed analysis of cause-effect relationships without overloading individual respondents with an excessively long questionnaire, reducing survey fatigue and increasing response quality.”*

**4. Description of Coding Plans for Open-Ended Questions**

**Reviewer’s Comment:** The coding plans mentioned for open-ended questions should be described.

**Response:** We have clarified that all open-ended questions were converted into numeric data. A brief description of the coding process has been added to the revised manuscript.

*“*Open-ended questions were transcribed using CATI software (e.g. https://www.b2binternational.com/research/methods/faq/what-is-cati/), converted into numeric data, capturing 100% of the responses. *”*

**5. Specification of Software Used for Data Analysis**

**Reviewer’s Comment:** Could you specify the software used for data analysis?

**Response:** Data coding of all variables was conducted using SPSS Statistics Version 28.0.1.0. This information has been explicitly stated in the revised manuscript.

**6. Details on the Pilot Phase**

**Reviewer’s Comment:** Please provide more details about the pilot phase—like how many participants were involved, and what specific changes were made based on the pilot?

**Response:** The pilot study involved 6 participants from the target demographic (18-34 years old). The objectives of the pilot were:

- Assessing the clarity and comprehensibility of survey questions.
- Estimating the expected duration of interviews to ensure participant engagement and minimize survey fatigue.
- Identifying any technical issues with the CATI system.
- Testing the logical flow of questionnaire items.

Based on feedback from the pilot study, minor wording adjustments were made to improve question clarity, and the estimated duration was confirmed to be within an acceptable range. These details have been added to the revised manuscript.

*“The pilot study, conducted with six participants aged 18-34, aimed to assess survey question clarity, estimate interview duration to maintain engagement, identify technical issues with the Computer Assisted Telephone Interviewing (CATI) script, and evaluate the logical flow of questionnaire items. Based on participant feedback, minor wording adjustments were made to improve clarity, and the estimated duration was confirmed to be within an acceptable range, leading to the development of the final questionnaire.”*

 We are grateful for your valuable feedback, which has helped us strengthen our manuscript. We believe that the revisions enhance the clarity and rigor of our study. Thank you for your thoughtful review.
